# Leveraging graph topology and semantic context for pharmacovigilance through twitter-streams

**DOI:** 10.1186/s12859-016-1220-5

**Published:** 2016-10-06

**Authors:** Ryan Eshleman, Rahul Singh

**Affiliations:** 1 Department of Computer Science, San Francisco State University, San Francisco, CA 94132 USA; 2 Center for Discovery and Innovation in Parasitic Diseases, University of California, San Diego, USA

**Keywords:** Pharmacovigilance, Pharmacology, Biological Modeling, Social Media, Text Mining

## Abstract

**Background:**

Adverse drug events (ADEs) constitute one of the leading causes of post-therapeutic death and their identification constitutes an important challenge of modern precision medicine. Unfortunately, the onset and effects of ADEs are often underreported complicating timely intervention. At over 500 million posts per day, Twitter is a commonly used social media platform. The ubiquity of day-to-day personal information exchange on Twitter makes it a promising target for data mining for ADE identification and intervention. Three technical challenges are central to this problem: (1) identification of salient medical keywords in (noisy) tweets, (2) mapping drug-effect relationships, and (3) classification of such relationships as adverse or non-adverse.

**Methods:**

We use a bipartite graph-theoretic representation called a drug-effect graph (DEG) for modeling drug and side effect relationships by representing the drugs and side effects as vertices. We construct individual DEGs on two data sources. The first DEG is constructed from the drug-effect relationships found in FDA package inserts as recorded in the SIDER database. The second DEG is constructed by mining the history of Twitter users. We use dictionary-based information extraction to identify medically-relevant concepts in tweets. Drugs, along with co-occurring symptoms are connected with edges weighted by temporal distance and frequency. Finally, information from the SIDER DEG is integrate with the Twitter DEG and edges are classified as either adverse or non-adverse using supervised machine learning.

**Results:**

We examine both graph-theoretic and semantic features for the classification task. The proposed approach can identify adverse drug effects with high accuracy with precision exceeding 85 % and F1 exceeding 81 %. When compared with leading methods at the state-of-the-art, which employ un-enriched graph-theoretic analysis alone, our method leads to improvements ranging between 5 and 8 % in terms of the aforementioned measures. Additionally, we employ our method to discover several ADEs which, though present in medical literature and Twitter-streams, are not represented in the SIDER databases.

**Conclusions:**

We present a DEG integration model as a powerful formalism for the analysis of drug-effect relationships that is general enough to accommodate diverse data sources, yet rigorous enough to provide a strong mechanism for ADE identification.

**Electronic supplementary material:**

The online version of this article (doi:10.1186/s12859-016-1220-5) contains supplementary material, which is available to authorized users.

## Background

### Introduction

The advent and proliferation of public social media platforms has provided unprecedented access to large streams of information related to drug effects. Such data can provide significant insights about drug action at a scale (both numeric and in terms of patient variability) that far outstrips what can often be considered in clinical trials. Consider for example this (real) Twitter message: “*Prednisone did kill most of my gut microbes & worsened my malnutrition though.*” Evidently, the tweet contains significant drug effect and adverse side-effect information. Furthermore, the information is being provided, possibly in a time-frame that is recent vis-à-vis the described drug-effects. Thanks to the “share all” usage promoted on social media, the content of such streams is widely available and may be used for pharmacovigilance and/or for collecting large-scale longitudinal information on therapeutics and their intended as well as side effects. At the same time, it should be noted that in the above tweet, the relevant information is embedded in a very short and noisy stream of conversations.

A particularly important formulation in this context is the identification of Adverse Drug Events/Effects (ADEs). An ADE can be defined as “any undesirable effect of a drug beyond its anticipated therapeutic effects occurring during clinical use” [1]. ADEs are estimated to account for 4.7 % of hospitalizations each year with an additional 6.7 % incidence rate among already hospitalized patients. Significantly, ADEs are between the fourth and sixth leading cause of death in hospitals [2]. ADEs have led to the withdrawal of several drugs, including Rofecoxib (Vioxx) whose long term cardiovascular risks were only later known [3]. Typically, for a given drug, it is expected that ADEs would be identified during clinical trials. However, due to a variety of factors including intrinsic polypharmacology of a drug, formulation of the trial criteria, and the limited diversity of the cohort that can be accommodated, ADEs may only be partially identified. Traditionally, aftermarket ADE identification is performed as a post-factum analysis of explicit event reports from voluntary post-market surveillance programs such as the US Food and Drug Administration’s Adverse Event Reporting System (FAERS) which allows clinicians, drug manufacturers and consumers to report potential ADEs. The shortcomings of such a spontaneous reporting systems (SRS) are well documented [4], and include latency, both in the reporting of ADEs and their analysis. More importantly, because reports are filed voluntarily by the users of the system, it is estimated that less than 10 % of cases involving ADEs actually get reported [5, 6].

Due to these reasons, identification of ADEs through algorithmic analysis of information from alternative sources such as electronic medical records, search engine query logs, internet forums and social media is increasingly becoming important. At over 500 million posts per day, Twitter is one of the more commonly used social media platforms. The ubiquity of its day-to-day personal information exchange model makes Twitter data (tweets) a promising target for ADE mining and real time pharmacovigilance. In this paper we describe an analytic framework for the identification of ADEs in Twitter-streams. The proposed approach leverages a graph-theoretic framework to represent drug and side effect information from tweets. This information is enriched using supplemental databases, such as the Side Effect Resource (SIDER) [7], that store ADE-related data. Information enrichment between the two sources is facilitated by an edge matching strategy.

The benefits of such a framework are threefold: first, it provides a mechanism for hypothesis generation of previously unreported ADEs. Second, the identification of known ADEs in real-time social media streams can promote timely medical intervention. Third, it can supplement current SRSs for a more accurate representation of ADE frequency in the public. The Key features and contributions of our work include:

- *Drug-Effect Knowledge Representation Framework*: A graph-theoretic knowledge representation framework which we call the Drug Effect Graph (DEG) is proposed to rigorously integrate and model drug and side effect relationships found in both Twitter-streams and static ADE databases. Our method shows performance gains of up to 8 % over previously published methods.
- *Automated Twitter-stream processing*: We implement a Twitter processing pipeline in two phases. First, we use a machine learned Twitter content filter to identify Twitter posts that relate a user’s personal experience. We then extract medically relevant terms with the MetaMap information extraction system.
- *Semantic Context of Medical Terms*: We incorporate semantic, conversation level features into the DEG model, including sentiment polarity and topic context. This allows the model to consider how the medical terms are being discussed when identifying ADEs, leading to significant performance gains.
- *Adverse Drug Effect Discovery*: The DEG model provides a mechanism for hypothesizing implicit ADEs that are not represented in the source data through edge enumeration and classification. Specifically, we identify several instances of ADEs which, though not present in the SIDER resource show evidence in both medical literature and the Twitter-stream.

### Prior work

Traditionally, ADEs have been identified through expert review of the event reports. Obvious scalability concerns have led to the application of data mining approaches on SRS repositories to find abnormally frequent drug-event relationships at scale. The FDA has employed Gamma Poisson Shrinking (GPS) and Multi-Item Gamma Poisson Shrinking (MGPS) algorithms [8] and the World Health Organization has used Bayesian Confidence Propagation Neural Networks (BCPNN) at its Upsalla Monitoring Center [9]. Additionally, empirical Bayes screening [10], odds ratios [11], and incidence report ratios [12] have been successfully implemented. Association rule mining [13] and biclustering [14] have also been explored to detect pairs of events that occur together with high confidence in a report database. These data sources have also been used to identify novel drug-drug interactions through regression modelling [15]. All of these methods, however, rely on the integrity and completeness of the voluntary reporting mechanism and therefore suffer from the issues mentioned previously.

Less direct methods of discovery have been explored to overcome the weaknesses of SRS analysis. For example, latent ADEs have been mined from patient Electronic Medical Records (EMRs) where prescriptions and symptoms are recorded individually, yet correlations between them have not been explicitly noted. EMRs contain both structured data (for example ICD9 codes) and unstructured data (clinical free text), both of which have been targeted for ADE discovery. Methods from SRS mining have been applied here such as GPS, MGPS, and BCPNN as well as association rule mining and biclustering approaches [16]. Information Extraction techniques from the field of Natural Language Processing have been used to identify drug-effect pairs in textual patient discharge summaries [17] with a *χ* ^2^ test to identify potential ADEs.

As an alternative to post-factum report analysis, predictive methods have been developed to leverage the content of wider biological resources. The FDA approved drug labels include textual pharmacological content such as the known drug side effects and indications. In this context, topic modelling has been applied to find common pharmacological “topics” or distributions of effects amongst the drug labels. These topics allow for drug clustering and prediction of unreported ADEs through association [18]. This method has also been applied to the question of drug repositioning. The mechanistic relationship between drug effects and drug-protein interactions motivated the integration of drug-protein interaction networks with the drug-effect networks implicit in drug labels to predict ADEs [19]. In a similar vein, off target drug interactions were predicted using the molecular similarity between drugs and known ligands. These predicted off-target interactions were shown to explain several known ADEs [20].

New and non traditional data sources have recently been explored as targets for ADE identification. These include search engine query logs, internet forums, and social media messages. With the hypothesis that users search for their symptoms and the drugs they are taking, the search logs of several major search engines were mined for drug-effect associations and drug pair-effect associations in [21]. As a proof of concept, the study showed the ability of search logs analysis to correctly correlate the drug pair Pravastatin and Paroxetine with symptoms of hyperglycemia. Unfortunately, access to search engine query logs is not a privilege afforded to most researchers. Additionally, association rules have been mined from these new media sources. An association rule is a rule of the form {*a*_*1*_ *,a*_*2,*_ *…,a*_*n*_} → {*c*_*1*_ *,c*_*2*_ *,…,c*_*m*_} where the occurrence of the antecedents *a*_*i*_ predict the occurrence of the consequents *c*_*j*_ with a given confidence. Association rule mining suffers from the lack of a global model so information about entities not present in the rule makes no contribution. Also, in the case of sparse data, confidence values may be over estimated. A related notion is that of language templates that represent usage patterns containing ADE references, for example “*I took* [*drug*]*, now I feel* [*effect*]*.*” This approach has been applied to text from the medical internet forum DailyStrength [22]. In addition to limitations related to association rules, the method is sensitive to minor variations in language. Furthermore, all ADE information are required to be contained within a single sentence precluding identification of ADEs with long range dependencies, for example across sentences in a user post, or across posts in a user’s history.

Twitter constitutes a natural target for ADE identification. In [23], a multi stage SVM classification pipeline was used in conjunction with an entity extraction system to identify individual tweets containing ADEs. This and related approaches [24–26] suffer from the same assumption as the forum mining method above, namely, that an individual tweet must contain all elements of the ADE. To address this issue, a global knowledge structure was proposed in [27] to represent co-occurrences of drug and effect references on social media as a bipartite graph and the ADE identification problem was posed as an edge classification problem between nodes in the graph. Such a graph representation can be understood as a generalization of the association rule approach for the single precedent – single consequent case, in that, a rule is represented as an edge in the graph. While clearly an advancement in holistic knowledge modeling, the implementation in [27] neither leveraged the inherent transferability of knowledge provided by the graph data structure nor did it attempt to exploit the context in which the drug and effect references were embedded. Obviously, the manner in which drugs and effects are discussed can provide valuable information about their relationship. The notion of enriching a relationship graph with semantic context has been successfully employed in the context of biomedical literature mining [28, 29].

The proposed method leverages the global drug-effect knowledge structure of the DEG model but does not limit it to the drugs and effects found in one data source. The formal DEG structure allows for an implicit mapping between edges in DEGs constructed from different data sources. We exploit this property by constructing two DEGs, one from drugs and effects identified in Twitter-streams and one from the Side Effect Resource (SIDER), and leveraging them jointly for ADE identification. Additionally, we enrich the DEG with information about the semantic context in which the drugs and effects are discussed on Twitter. Overall, the method allows for the identification of ADEs in social media with long range dependencies. As a framework for general drug – side effect analysis, it is not strictly limited to use on Twitter and SIDER. It may be applied to other social media platforms such as forums and blogs as well as other static knowledge bases. Thus, the data sources can be broader and more current than those used in traditional SRS and EMR based approaches. Finally, we use the DEG model to hypothesize unrepresented ADEs by enumerating and individually classifying all potential edges in the DEG. *In particular, we are able to identify several ADEs that are not reported in the SIDER resource.*

## Methods

Our method is based on a graph-theoretic framework, which we call the Drug Effect Graph (DEG). A DEG is a graph that models the relationships between drugs and side effects. Properties of DEG(s) can be used to distinguish between relationships that represent ADEs and those that do not. In this work we construct two DEGs over two datasets and then use them jointly to improve overall performance of ADE identification. The first DEG is built on the drug - side effect relationships recorded in the Side Effect Resource (SIDER) [7] and the second DEG is built by extracting information from the social media network Twitter. Finally, we use the DEG knowledge structure to discover ADEs that, while explicitly absent in the data source, are otherwise noted in medical literature.

### Data sources

Our work is built on publically available data from two resources, the SIDER Side Effect Resource and the Twitter microblog social media platform. SIDER is a database of drug and side effect relationships extracted from the text of FDA package inserts that list known drug side effects. This database currently contains 1,430 drugs, 5,868 side effects, and 139,756 drug-side effect relationships. Drugs are identified by their PubChem Compound ID, thus individual chemical compounds with multiple trade names are identified by a single ID. Side effect terms come from the Medical Dictionary for Regulatory Activities (MedDRa) list and are provided with concept identifiers that map to the Unified Medical Language System (UMLS) [30].

Twitter is a microblog social networking platform that allows users to post “status updates,” or “tweets,” containing up to 140 characters of Unicode text. Users are neither limited in post frequency nor censored on content. Twitter data is accessed through two free public APIs. The streaming API provides access to the real-time Twitter-stream, and the search API allows querying against previously posted tweets. We note that neither of these public APIs guarantee completeness of results, and each API provides access to only a sampling of the total posts reported at approximately 1 %.

### Basic concepts and definitions

This section presents the set of definitions and notations used throughout the paper. *Drugs* refer to chemicals used for therapeutic intervention and are limited here to the set of drugs contained in the SIDER database and associated synonyms. We understand *side effect* to be any physiological effect caused by taking the drug, excluding the expected therapeutic effect. Side effects are a consequence of drug polypharmacology as well the interplay of bio-molecules and pathways. The set of side effects we consider is limited to those contained in the SIDER database. A *Drug Effect Graph (DEG)* is a bipartite graph modeling drug-side effect relationships where drugs and side effects are disjoint partite sets of nodes and an edge indicates a drug - side effect relationship. *DEG*_*Sider*_ is a DEG constructed on the SIDER database. *DEG*_*user*_ is a DEG constructed out of the drug and effect entities found in the tweet history of an individual Twitter user. *DEG*_*Twitter*_ is a DEG constructed out of one or more *DEG*_*User*_ graphs by combining their nodes and edges. An *adverse edge* is an edge in a DEG (*DEG*_*Sider*_, *DEG*_*User*_ *, or DEG*_*Twitter*_) that connects a *drug* to an *effect* known to be adverse in the SIDER database. A *pseudo-non-adverse* edge is any edge that is not explicitly considered *adverse*. The *pharmacological neighborhood* of a drug node *x* in a DEG is denoted by *Γ*(*x*) and is defined as the set of nodes that can be reached within two hops of *x*. Thus, two drugs are considered to be pharmacological neighbors if they share a side effect and vice versa.

We consider a tweet to be *experiential* if it relates the personal experience of an individual user, as opposed to tweets posted as advertisements, news sources or other non personal tweets. An *entity* in a tweet is any drug or drug-effect mentioned within the tweet.

### Method overview

**Fig. 1:**
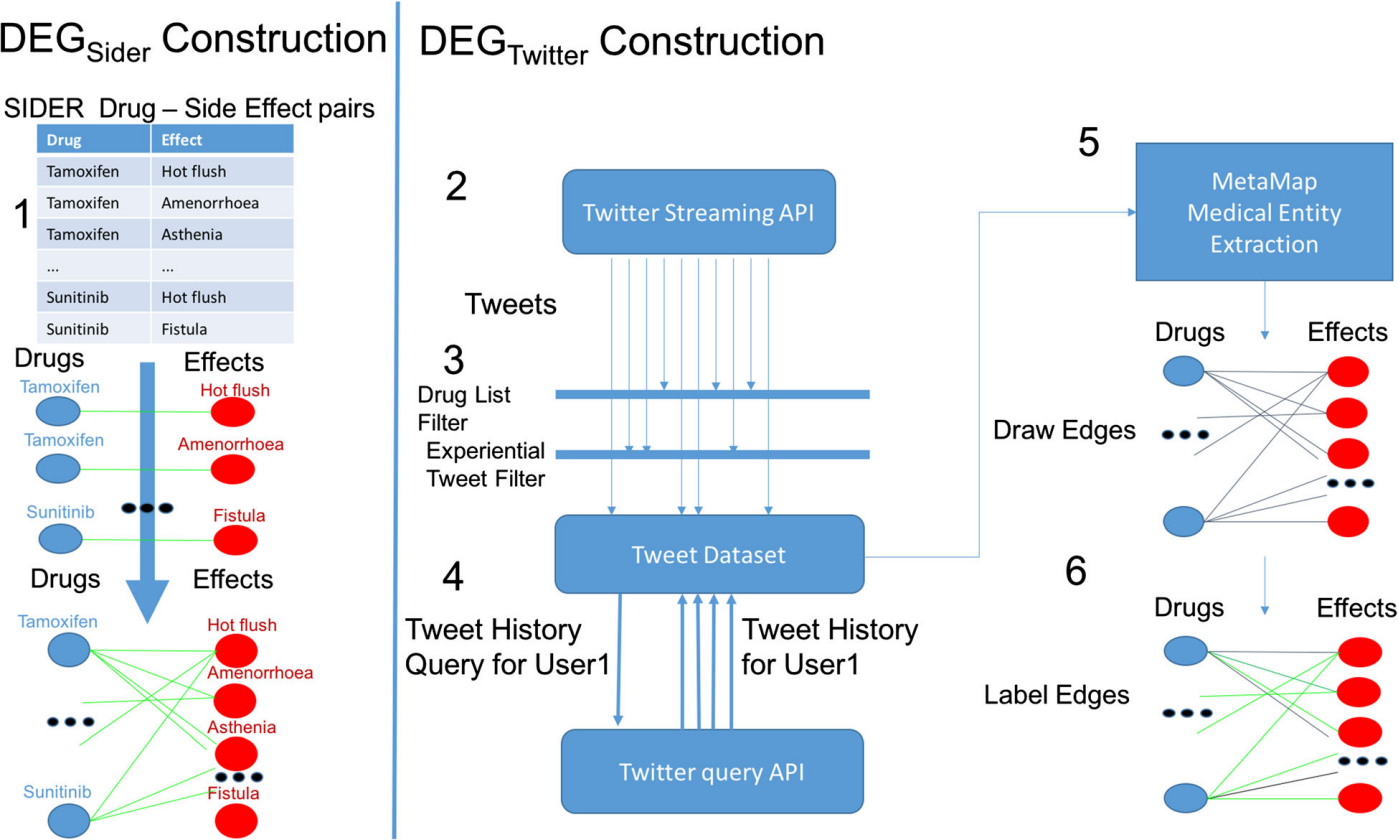
Method Overview. Two DEGs are constructed from two datasets, SIDER (left) and Twitter (Right) 1. DEG_Sider_ is a graph built from the drug and effect relationships recorded in the SIDER database, forming a comprehensive DEG containing the Adverse Drug Relationships known to SIDER. 2. DEG_Twitter_ is constructed by mining Twitter posts for mentions of drugs and effects. 3. The live Twitter-stream is filtered for tweets that reference drugs and relate an individual user’s experience. 4. From the tweets gathered from the Twitter-stream, user’s histories are queried. 5. Drug and effect mentions are extracted with MetaMap. 6. An edge is drawn between a drug and an effect if they are both referenced in a user’s history within a fixed window of time. These edges are labeled as adverse or psuedo-non-adverse based on their presence or absence in SIDER

Our method consists of the construction of two DEGs, *DEG*_*Sider*_ and *DEG*_*Twitter*_, that are used in conjunction to identify ADE’s in Twitter-streams. Figure 1 provides an overview of the DEG construction process. The construction of *DEG*_*Sider*_ is relatively straightforward; the nodes are all individual drugs and effects in SIDER and the drug-effect relationships from SIDER are represented as edges. The construction of *DEG*_*Twitter*_ is significantly more complex. First, a set of tweets is collected using the Twitter streaming API. We filter for tweets that are both *experiential* and reference a drug from a list of 200 commonly prescribed drugs [Additional file 1: Table S1]. User tweet histories are then queried using the Twitter query API. Drug and effect *entities* are extracted from the user tweet histories with the MetaMap information extraction system. Edges are constructed in the *DEG*_*Twitter*_ between drug and effect entities that are mentioned within a prescribed time window in an individual user’s history. Edges are labeled based on the SIDER database. These DEGs are used to build classification models that can, with high accuracy, predict whether a drug-effect relationship is adverse.

### Constructing *DEG*_*sider*_

The SIDER database contains a set of drugs *D* = {*d*_*1*_ *,d*_*2*_ *,…d*_*n*_}, where *n =* 1,430, a set of side effects *S = {s*_*1*_ *,s*_*2*_ *,…,s*_*m*_ *}* with *m* = 5,868, and a set of relationships *R = {r*_*1*_ *,r*_*2*_ *,…r*_*k*_ *}*, *where r*_*i*_ ∈ *D × S* and *k =* 139,756. Because *R* contains no (*drug*, *drug*) or (*side effect, side effect*) pairs, we construct a DEG by defining a bipartite graph *DEG*_*sider*_ = {*U*, *V*, *E*} where *U* = *D* and *V* = *S*, with the constraint: *U ∩ V = ∅* and the edges in *E* capture the drug-side effect relationships and take the form (*d*, *e*) with *d* ∈ *U* and *e* ∈ *V*. Figure 1(1) illustrates the construction of *DEG*_*Sider*_.

### Topological features

Our goal, is to build a model based on the topological properties of *DEG*_*sider*_ that can differentiate between edges that represent adverse effects and those that do not.

The first and simplest feature that we consider in classifying an edge *(d,e)* is the number of edges incident to each node, or the edge degrees. Next, we consider the number of common neighbors for each edge, as given by Eq. (1),

1 $$ N\ \left(d,e\right) = \left|\ \varGamma (d){\displaystyle \cap}\varGamma (e)\ \right|. $$

**Fig. 2:**
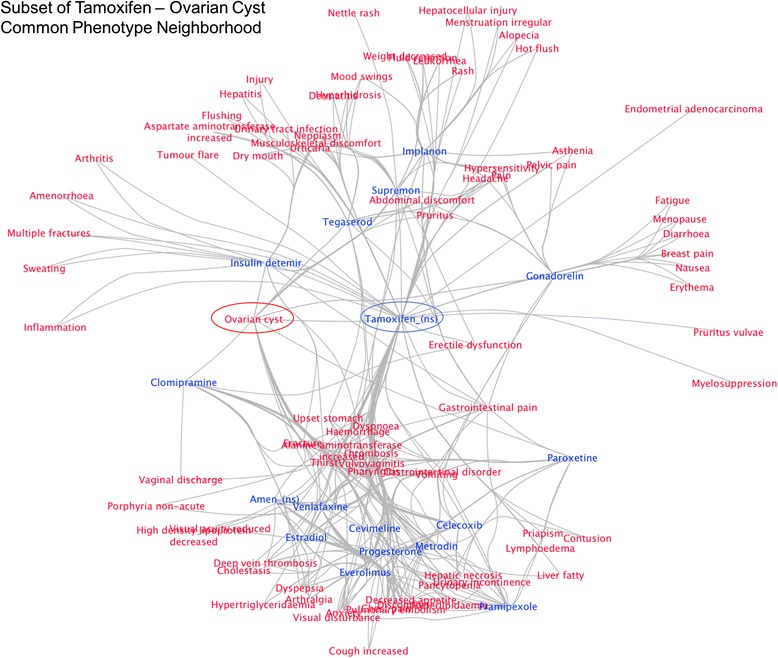
A subset of the Common Neighborhood Sub Network of the drug Tamoxifen and effect Ovarian cyst. This network defines a pharmacological neighborhood where every node can reach every other node within a maximum of three hops, Tamoxifen and Ovarian cyst serve as hub nodes that can connect any two other nodes. The size of a pharmacological neighborhood is used as a measure of relatedness between the two hub nodes

The common neighborhood of an edge *(d,e)* defines a connected sub-graph of *DEG*_*sider*_ centered around *(d,e),* where the maximum distance between any two nodes from the same set (ie, two drug nodes) is two hops, and the maximum distance between nodes of a different set is three hops. The intuition behind this measure is that the size of this sub-graph indicates a level of relatedness. The larger the neighborhood is, the more drug and effect neighbors the two nodes have in common. For example, an edge that connects a drug that is known to have many gastrointestinal side effects with a gastrointestinal side effect will have a larger common neighborhood than if the drug were connected to a neurological side effect. Figure 2 shows a portion of the common neighborhood sub network for the drug *Tamoxifen* (a breast cancer drug) and the side effect *Ovarian Cyst*.

The common neighbor size feature is not normalized and may be highly influenced by the connectedness of the nodes in *(d,e)* and not adequately measure the neighborhood similarity. To account for this fact, we compute another measure that normalizes the common neighborhood size by the size of the combined neighborhoods. This measures the similarity of the two neighborhoods and is computed using Eq. (2),

2 $$ S\left(d,e\right) = \frac{\left|\varGamma (d)\ {\displaystyle \cap }\ \varGamma (e)\ \right|}{\left|\ \varGamma (d)\ {\displaystyle \cup }\ \varGamma (e)\ \right|} $$

Eq.(2) reflects the proportion of the total neighborhood (bottom term) that is also in the common neighborhood which is equivalent to the Jaccard Coefficient. A value of 1 implies that *Γ*(*d*) = *Γ*(*e*). Continuing with this logic, it is apparent that the neighborhood of a node is also influenced by the individual connectedness of the neighborhood member nodes. For example, *Headache* is a very common side effect, and its presence in the neighborhood may be less informative because it is present in many neighborhoods. To address this, we adopt the Adamic-Adar index [31] defined in Eq. (3):

3 $$ A\left(d,e\right) = {\displaystyle \sum_{z\in \varGamma (d)\ {\displaystyle \cap }\ \varGamma (e)}}\frac{1}{log\left|\ \varGamma (z)\ \right|} $$

This equation discounts a common neighborhood node’s importance by the size of its individual neighborhood, this approach has a corollary in the inverse document frequency measure from text analysis. The Adamic-Adar index formulation implies that the connectedness of individual nodes and by extension edges, is informative in itself. To capture the ‘connectedness’ of an edge, we take the notion of ‘preferential attachment’ from [32]. Preferential attachment is motived by the property of many biological (and scale free) networks, that a node is more likely to be linked to a node that already has a large number of links than one with few links. This notion is captured using Eq. (4).

4 $$ PA\left(d,e\right)=\left|\varGamma (d)\right|*\left|\varGamma (e)\right| $$

In sum, the aforementioned topological features form a numerical representation of the graph neighborhood characteristics that surround a given edge in a DEG.

### Constructing *DEG*_*Twitter*_

*DEG*_*Twitter*_ is a DEG designed to model the drug and side effect relationships as they are expressed by Twitter users. The *DEG*_*Twitter*_ construction is performed in four steps, similar to the method described in [27]. First, we identify users whose Twitter status updates include drug references. Second, we obtain the recent tweet history from these users. Third, drug and effect references are extracted from the user histories. Finally, edges are drawn between drugs and effects based on temporal proximity.

The user identification step is performed through the Twitter streaming API which provides access to a sampling of the live tweets and the option to set filters for specified keywords. We recorded tweets over two weeks between January 28, 2016 and February 11, 2016 filtering for 200 of the most commonly prescribed drugs [Additional file 1: Table S1]. Our filtering led to shortlisting of 157,735 tweets from 7981 users. From this set, the most recent tweets from each user were queried, with a limit of 400 tweets per user. In the next step, tweet filtering was employed to limit our data set to only those tweets that relate a user’s personal experience, or *experiential* tweets. We formulate tweet filtering as the binary classification task of labeling a tweet *experiential* or *non-experiential* and use a supervised classifier (Random Forests) learned on a dataset of 1500 tweets manually labeled for this purpose. The features extracted from these tweets are enumerated in Table 1 and are similar to the features suggested in [23]. Evaluation of the classification performance is provided in the *Results* section.

**Table 1:** Text Features used for Tweet Classification

	Tweet Text Feature
1	Number of hashtags
2	Number of words indicating negation
3	Number of URLs
4	Number of pronouns
5	Number of drug entities
6	Number of effect entities
7	Bag of words text representation

Table 1 The textual features extracted from twitter text for classification. Many spam and irrelevant tweets are designed to redirect a user to a desired website, thus the presence of URL’s in the tweet text is a strong indicator of spam. Spam tweets often contain many hashtags in an attempt to exploit trending topics, this makes the number of hashtags a very informative feature as well

Extraction of drug and side effect entities from Twitter text is performed by using the MetaMap biomedical annotator [33] which provides mappings from plain text to terms in the Unified Medical Language System (UMLS). MetaMap was designed for use on biomedical research literature. However, language usage in the medical domain can be distinct from usage on Twitter, which can result in incorrect mappings. We encountered significant problems with medical acronyms and abbreviations that commonly have different meanings on Twitter. For example, the characters “pic” is a common abbreviation of the word “picture” on Twitter, but “PIC” is also a medical acronym for “Punctate Inner Choroidopathy.” This is one of many such examples that required us to manually curate a stop list of acronyms, abbreviations and terms that otherwise cause frequent erroneous mappings [Additional file 1: Table S2]. Entities appearing in this stop list were disregarded. An additional difficulty in entity identification is the proliferation of synonyms and trade names that refer to the same chemical compound. For example, there are well over 100 trade names for Ibuprofen, including Advil, Motrin, and Nurofen, many of these synonyms have unique entries in the UMLS. To overcome this, we use the drug synonym resources available through the PubChem [34] API which aggregates synonym lists from several sources, including the Medical Subject Heading (MeSH) vocabulary, Depositor supplied terms, and other chemical databases. We use this list to resolve synonymous references to a canonical term.

After the tweet dataset has been filtered for *experiential* tweets and the medical entities identified, the *DEG*_*Twitter*_ is constructed in two steps. First individual user histories are used to construct a set of user DEGs or *DEG*_*user*_ graphs. Next, this set of *DEG*_*user*_ graphs is merged to form the *DEG*_*Twitter*_. To begin, individual users’ tweet histories are examined and edges are drawn between citations of drugs and side effects occurring within a fixed time window. This window length is an experimentally-set parameter and we examine its effect in the Results section. The edges are weighted by the average time between the drug and effect mentions. This produces a set of *DEG*_*user*_, graphs, *USERS* = {*DEG*_*user,1*_ *, DEG*_*user,2*_ *,…, DEG*_*user,n*_} where, in our case, *n* = 7,981, *DEG*_*user,i*_ = {*V*_*i*_ *,E*_*i*_}, and *V*_*i*_ and *E*_*i*_ are the nodes and edges found in the history of user *i* per the description above. The global *DEG*_*Twitter*_ is constructed by aggregating the user graphs, *DEG*_*Twitter*_ *= {V*_*Twitter*_ *,E*_*Twitter*_ *}* where *V*_*Twitter*_ = *U*_*V* ∈ *USERS*_ *V*, *E*_*Twitter*_ = *U*_*E* ∈ *USERS*_ *E* and the temporal edge weights are averaged for common edges. A second edge weight is added to the model for the frequency of the edge in *USERS*, or equivalently, t he number of users whose histories contain the same (*drug,effect*) pair.

### Case study: constructing *DEG*_*Twitter*_

**Fig. 3:**
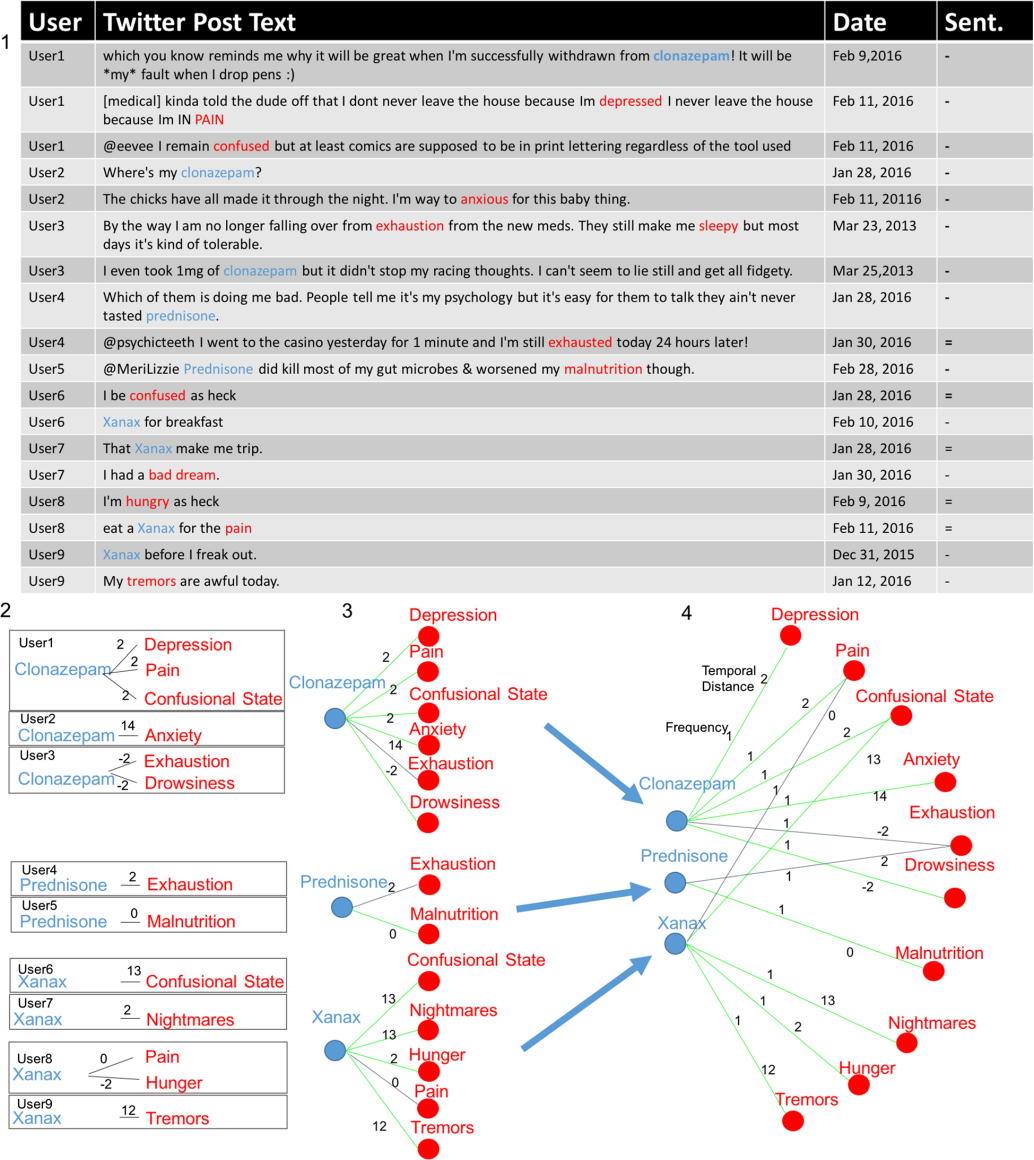
Illustration of DEG_Twitter_ construction. DEG_Twitter_ is constructed from the drugs and side effects mentions in user tweet histories. 1. 18 example tweets from 9 individual users from our dataset (after experiential tweet filtering and entity extraction). The table includes anonymized usernames, tweet text (nominally altered/censored where appropriate), date the tweet was posted, and the computed sentiment. Drug entities extracted from the text are highlighted in blue and effects in red. 2. Individual user histories are used to construct a set of DEG_User_ graphs. User graphs contain all entities identified in a user history that were posted within a specified distance of an entity of the opposite class (20 days in this case). A drug and a side effect have a connecting edge if they were mentioned within the window. Edges are weighted by temporal distance (in days). 3. Edges are labeled according to the SIDER database as either adverse (green) or pseudo-non-adverse (grey). 4. Edges are aggregated across all graphs forming the DEG_Twitter_ which contains all nodes and edges found in DEG_user_ graphs, temporal distance edge weights are averaged if two DEG_User_ graphs contained the same edge. An additional edge weight reflects the number of DEG_user_ graphs that contain the edge

To illustrate the above process, we present an example that demonstrates the construction of *DEG*_*Twitter*_ using 18 tweets from our dataset, shown in Fig. 3. These tweets were posted by a total of 9 unique users. User1 posted 3 tweets, User5 posted one tweet and the rest posted 2 tweets each. Each of these tweets successfully passed the *experiential* tweet filter and thus each related a user’s individual experience. References to drug and effect entities were identified with MetaMap. Three unique drugs were found: *Clonazepam*, *Prednisone*, and *Xanax*. Furthermore, the following nine effects were identified: *Depression*, *Pain*, *Confusional State*, *Anxiety*, *Exhaustion*, *Malnutrition*, *Nighmares*, *Hunger*, and, *Tremors.* These drug and effect mentions have been used to build the individual *DEG*_*user*_ graphs shown at label 2 in the Fig. 3. User1 mentioned *Clonazepam* on Feb. 9, 2016 and then mentioned *Depression*, *Pain*, and *Confusion* on February 11, 2016. Each effect was mentioned two days after *Clonazepam.* Accordingly, a four node graph is constructed with edges connecting each effect to *Clonazepam* with a weight of two. A similar operation is performed for every user, resulting in 9 individual *DEG*_*User*_ graphs.

Next, the edges in the DEG_User_ graphs are labeled per their presence in SIDER. For example, SIDER lists *Depression* as a known side effect of *Clonazepam*, so the edge is labeled adverse (colored green). Edges that do not appear in SIDER are labeled pseudo-non-adverse (colored grey). Label 3 in Fig. 3 shows both the edge labeling and the beginning of the merging step where drug nodes are merged between DEG_User_ instances. The merging step is completed by merging all effect nodes (label 4). Two edge weights are assigned to each edge to reflect average temporal distance and frequency. In this case, all frequency values are equal to 1. We now have an instance of *DEG*_*Twitter*_ that represents all of the drug-effect relationships mined from the set of tweets.

### Extracting semantic context

Drugs and effects mentioned in a tweet are embedded within a semantic context consisting of the words contained in the text of the tweet. We look to capture two elements of this context through sentiment analysis and topic modeling. The sentiment analysis of a text attempts to assign it a ‘sentiment polarity,’ that is, whether a tweet expresses positive, negative, or neutral sentiment. Such sentiment can help distinguish the role an entity is playing in a sentence. For example, the entity *headache* may be the symptom being treated when it is mentioned in a positive tweet, for example “*I love Advil! I no longer have a headache!*” However, the sentence “*I hate how caffeine always gives me a headache*” shows a negative sentiment and indicates that *headache* is a side effect.

To compute the sentiment context of a given edge, we apply sentiment analysis to all individual tweets contributing to the edge. The sentiment analysis function is provided by the Stanford Sentiment Analysis package [35] which performs a deep syntactic parse of the input text resulting in a grammatical parse tree. A neural network is then applied to recursively predict the sentiment of each node in the tree and node sentiments are aggregated to arrive at the final sentiment assignment of the text. Although this package was not designed specifically for use on Twitter text, which contains notoriously distinct language usage patterns, it was evaluated on Twitter text in [36] and it showed strong performance. This sentiment analysis package identifies a string of text as *Very Positive, Positive, Neutral, Negative, or Very Negative* which we assign the nominal values of 2, 1, 0, -1, and -2 respectively. The analysis thus takes the form *f*_*sentiment*_ : *tweet* → {2, 1, 0, − 1, − 2} and the sentiment context of an edge is a tuple (*S*_*d*_, *S*_*e*_). Where S_d_ is the average sentiment of the tweets that contribute to the edge and mention drug *d*, shown in Eq. (5), where tweets_e_ is the set of tweets contributing to the edge and containing the entity *e*.

5 $$ S(e)=\frac{{\displaystyle {\sum}_{tweet\in tweet{s}_e\ }}{f}_{sentiment}(tweet)}{\left| tweet{s}_e\right|}. $$

The second element of the semantic context that we consider is the topic context. We do this through Latent Dirichlet Allocation (LDA) topic modelling which models a document as a distribution over a set of topics, *P(θ),* and a topic as a distribution of words, *P(w|θ)*. LDA describes a generative process of document formation, where the words in a document are generated in two steps: First, a topic is chosen from the topic distribution *P(θ = z),* where *z* is the topic. Next, a word *w* is chosen from the word distributions of topic *z*, *P(w|θ = z).* Accordingly, the word distributions in a document are prescribed in Eq. (6),

6 $$ P\left(w\Big|\theta \right)={\displaystyle \sum_z}P\left(w\Big|z\right)P\left(z\Big|\theta \right). $$

This formalism lets us place entities within the context of topics being discussed on Twitter. We use the LDA optimization method described in [37] which is based on an online variational Bayes algorithm with *P(θ)* and *P(w|θ)* computed over all tweets containing drug or effect references. We define the topic vector of a tweet *θ*_*tweet*_ to be a *k*-vector whose indices sum to 1, where *k* is the number of topics. *k* is an experimental parameter we have set to 20 for our experiments. The entity topic context is the average vector of all tweets containing the entity, Eq. (7),

7 $$ c(e)=\frac{{\displaystyle {\sum}_{tweet\in tweet{s}_e\ }}{\theta}_{tweet}}{\left| tweet{s}_e\right|}. $$

### Enriching *DEG*_*Twitter*_ with *DEG*_*Sider*_

Both *DEG*_*Twitter*_ and *DEG*_*Sider*_ capture important, yet potentially distinct aspects of drug effect relationships. To incorporate this information in a single representation, we combine the features as follows. Let *features*(*DEG*_*Sider*_ , *edge*) denote the function which given an edge generates the aforementioned topological features from *DEG*_*Sider*_. Similarly, let *features*(*DEG*_*Twitter*_ , *edge*) generate the features from the *DEG*_*Twitter*_ including both topological and semantic context features. We define the enriched feature set as the union of *DEG*_*Sider*_ and *DEG*_*Twitter*_ features, as described in Eq. (8)

8 $$ enriched\_ features(edge)=\Big(\left( features\left(DE{G}_{Sider}, edge\right)\ {\displaystyle \cup }\  features\left(DE{G}_{Twitter}, edge\right)\right) $$

### Adverse drug effect discovery

*DEG*_*Sider*_ serves as a comprehensive representation of the known ADEs (we will see in *Results* section that *DEG*_*Twitter*_ represents a smaller subset of the known ADEs). As such, we use the *DEG*_*Sider*_ to hypothesize the existence of unrepresented ADEs by enumerating all possible edges *E*_*a*_ = *D × S* where *D* and *S* is the sets of drugs and effects in *DEG*_*sider*_ respectively. The edges already present in *DEG*_*Sider*_ are removed from *E*_*a*_ creating the set of all possible hypothetical edges *E*_*h*_ = *E*_*a*_ \ *E*_*s*_ where *E*_*s*_ is the set of edges in *DEG*_*Sider*._ Each edge in *E*_*h*_ is individually inserted in to *DEG*_*Sider*_ and then classified.

After classifying the hypothetical edges, we look for evidence in the Twitter-stream of the edges that were classified as *adverse*. We used the Twitter query API to perform a search of the Twitter-streams for users who mention both the drug and the side effect within a 20-day window. We query the API for 100 tweets that mention the drug and then request the 400 most recent tweets from each user. The medical entities are extracted from the tweet text to identify the hypothesized ADE in question.

## Results

In this section, we assess the role of the individual design steps in the construction of the *DEG*_*Sider*_ *,* the *DEG*_*Twitter*_ and the enriched *DEG*_*Twitter*_. We also examine the results of our ADE hypothesis generation method. We begin by observing the network characteristics of *DEG*_*Sider*_ and investigating the ability of our topological features to capture the difference between adverse and pseudo-non-adverse edges. Next we look at the construction of *DEG*_*Twitter*_ and account for the effects of the experimental parameters on its network properties. We then evaluate the overall efficacy of our method’s ability to identify ADEs in the two DEGs and we measure the performance gains of our approach over previously published methods. Additionally, we identify several ADEs through our hypothesis generation method that are not present in the SIDER database, yet of which evidence is found both on Twitter and in the medical literature.

### *DEG*_*Sider*_ characteristics

The topological features described above were chosen for their ability to describe the relationship between a drug and a side effect based on measurements of their pharmacological neighborhood. Inherent in this formulation is the hypothesis that the distributions of these features vary between the sets of adverse and non-adverse edges. Because SIDER only provides examples of adverse edges, we generate a set of pseudo-non-adverse examples by randomly drawing edges in *DEG*_*Sider*_ that are not listed as adverse in SIDER. We generate a set of pseudo-non-adverse edges equal in size to the number of adverse edges in SIDER, yielding 139,756 pseudo-negative adverse edges for a total of 279,512 edges.

**Fig. 4:**
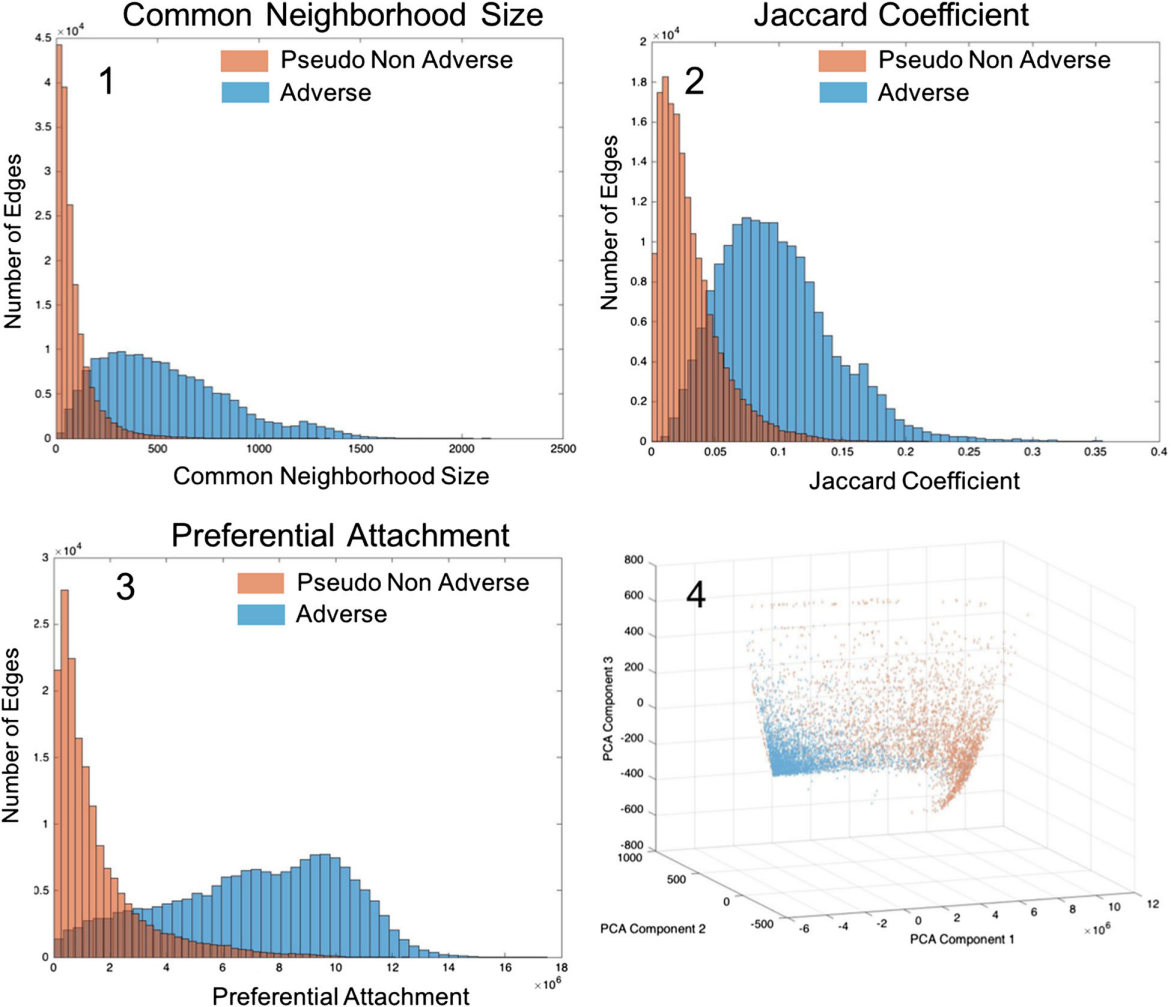
DEG_Sider_ feature distributions. The DEG_Sider_ topological feature distributions for Common Neighbor Size, Jaccard Coefficient, and Preferential Attachment (parts 1,2 and 3). In each case, the shape is distinct between the adverse and pseudo-non-adverse edges. Part 4 shows all features projected to the three most explanatory PCA components. As with individual features, each class takes a distinct shape

To examine the edge properties of each class of edges, histograms of the three most distinguishing features are plotted in Fig. 4 (1-3), revealing distinct distributions between classes. While there is some overlap between the two classes for each feature, this observation builds confidence in both the existence of topological differences between edge classes and our model’s ability to numerically represent it. We further obtain an aggregate view of the entire feature space through a lower dimensional projection using principal component analysis which is presented in Fig. 4 (4). Here we have a three dimensional rendering based on the first three components. The trend from the histograms continues and we see distinct distributions, albeit with some overlap, between classes of edges.

### Adverse drug effect classification in *DEG*_*Sider*_

We cast the ADE identification problem as a supervised learning task that requires a binary classification model to identify edges as either adverse or non-adverse based on the topological features derived from *DEG*_*Sider*_. The set of adverse and pseudo-non-adverse edges that compose *DEG*_*Sider*_ create a set of 279,512 edges evenly balanced between the two types. This set is randomly split into two sets of equal size, which composes our training and test sets. 10-fold cross validation is performed on the training set for parameter tuning. A classifier is then trained on the training set and its performance is measured on the test set. Results from these evaluations are provided in the *Results* section.

### *DEG*_*Twitter*_ construction

**Fig. 5:**
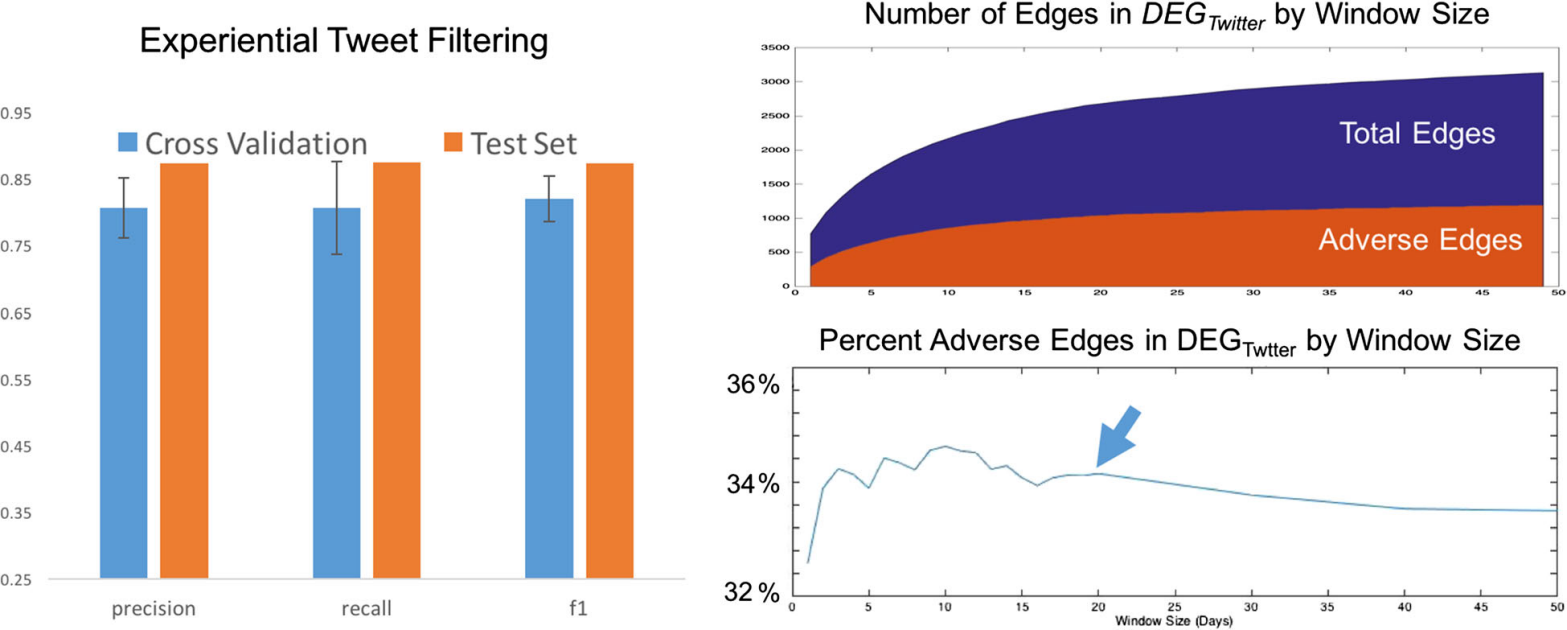
Tweet filtering and DEG_Twitter_ construction characteristics. Left, Performance evaluation results for experiential tweet filtering. Blue shows average 10-fold cross-validated results on the training set with error bars indicating one standard deviation above and below the mean. Orange is the result on the test set. Right, top shows growth rate of DEG_Twitter_ as the window size, t, increases (x axis). Y axis is number of edges. The number of adverse edges is shown in orange. Right, bottom shows the percentage of total edges that are adverse. The arrow indicates t = 20 where this value stabilizes and begins to slowly decline

Two important steps are involved in the construction of the *DEG*_*Twitter*_, pre-processing/tweet-filtering, and estimation of an appropriate time window parameter value. To evaluate our method’s ability to filter out non-experiential tweets, we sample 1500 tweets from the Twitter Streaming API that contain drug or effect mentions. These tweets are manually annotated as experiential or non-experiential and the textual features are extracted as described in Table 1 above. This data set is split into a training and a test set, containing 60 % and 40 % of the tweets respectively. Classification is performed with a Random Forests classifier (10 trees) and performance is evaluated with 10-fold cross validation on the training set. A classifier is then trained on the entire training set and its performance is evaluated on the test set. Three standard measures of performance are calculated, precision, recall and *F1*. Our method shows strong performance of > .85 on all measures in the test set. See Fig. 5, left. Encouraged by these results, we apply this classifier to remove irrelevant, non-experiential tweets from our dataset.

The construction of *DEG*_*Twitter*_ out of the cleaned dataset is governed by one experimental parameter *t*, which is the size of the time window used to filter out drug-effect mentions that exceed a temporal distance, measured in days. To determine the proper value, we look at its effect on the percentage of adverse edges in the DEG. We expect that as *t* grows too large, the percentage of adverse edges will at some point begin to drop because the loosening inclusion parameter will allow more spurious edges. In the limit, the ratio would settle to a value specific to the dataset being sampled. Figure 5, right, shows plots of the total edges and adverse edges (top), and of the percentage of total edges that are adverse (bottom) as *t* increases. The percentage adverse edges is relatively stable between *t* = *1* and *t* = 20, hovering around 34 % with a maximum at *t* = 10. However, at *t* = 20, the percentage stabilizes and steadily drops as *t* increases. Accordingly, we set the value to *t* = 20 days. The set of nodes in *DEG*_*Twitter*_ is unsurprisingly a subset of those in *DEG*_*Sider*_ because of the generally non-technical scope of the language and less precise usage found in Twitter posts. Accordingly, the *DEG*_*Twitter*_ contains 363 unique effects, 264 unique drugs and a total of 2780 edges.

It is worth noting at this point that if the ADE identification methods [23–26], requiring the drug and effect(s) to occur in the same tweet are employed instead, then the size of the dataset is severely diminished; only 44 edges are retained in *DEG*_*Twitter*_ – a number far too small to conduct analysis with. The proposed method, by contrast, allows accounting for significantly more data.

### *DEG*_*Sider*_ edge classification performance

**Fig. 6:**
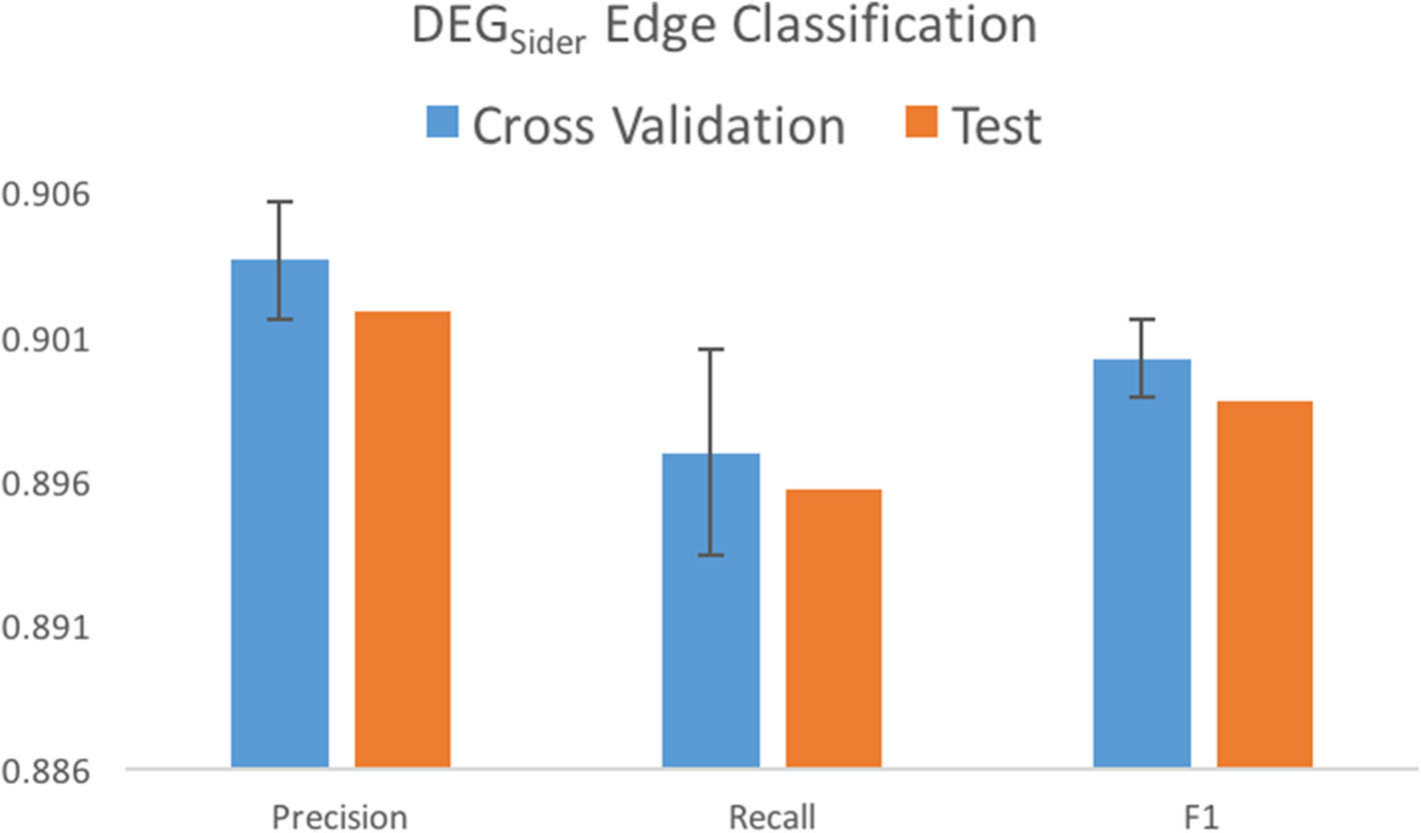
Performance evaluation of DEG_Sider_ edge classification. Mean 10 fold cross validation results shown in blue with error bars indicating one standard deviation above and below the mean. Performance on the test set are shown in orange

Classification of the adverse, pseudo-non-adverse edges in the *DEG*_*Sider*_ was performed with the Random Forests ensemble classifier (10 trees). The 279,512 *DEG*_*Sider*_ edges set was randomly partitioned into a training and test set of 139,756 edges each. The standard performance measurements of precision, recall and *F1* are recorded with results shown in Fig. 6. Evaluation on the training and test sets both showed very strong performance, indicating that our DEG model for ADE representation captures significant properties of drug and side effect relationships and that the distinctions between adverse and pseudo-non-adverse relationships are captured in these properties.

### *DEG*_*Twitter*_ and enriched *DEG*_*Twitter*_ edge classification performance

Previous research had considered only the topological features of the *DEG*_*Twitter*_. To measure the performance gains of our method over previous research, we evaluate classification performance on topological features alone and then incrementally add our novel features to the model and measure performance gains at each step. In total, four feature sets are evaluated: 1) Topological (baseline), 2) Topological and Sentiment features, 3) Topological, Sentiment, and Topic features, and 4) Topological, Sentiment, Topic, and *DEG*_*Sider*_ Enriched features.

**Fig. 7:**
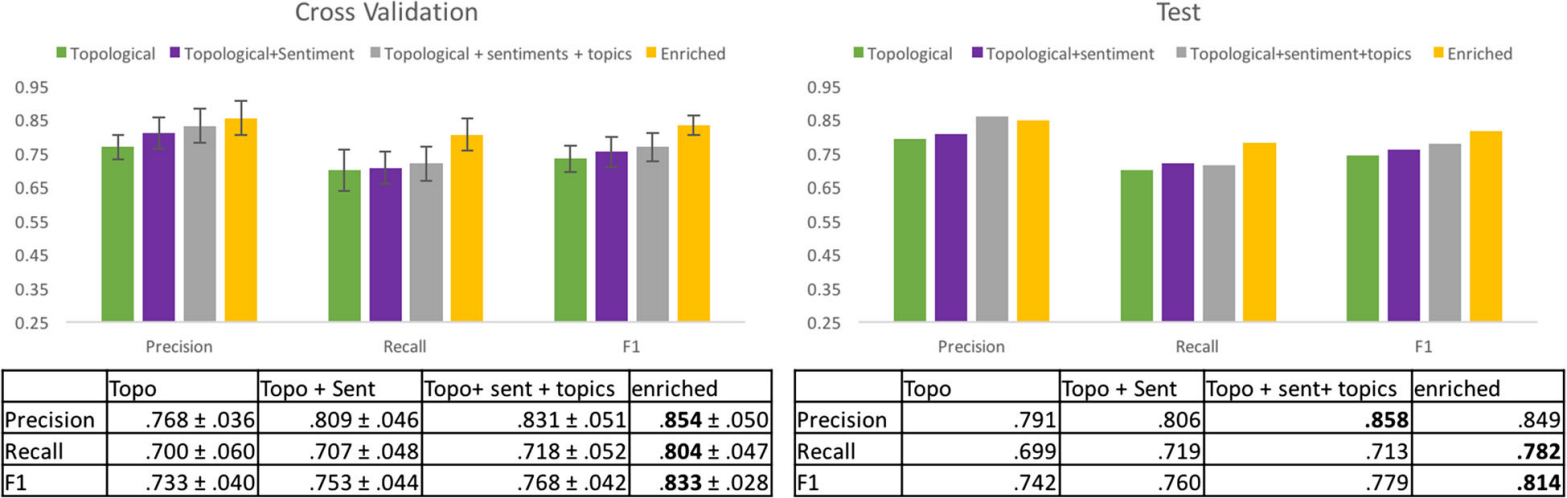
Performance evaluation results for DEG_Twitter_ as new features are added to the model. Left: Mean 10-fold cross validation results with errors bars indicating +/- 1 standard deviation. Actual values in table below Right: Performance results on the test set. Performance on the test set steadily increases with the expanding feature set. Biggest gains are achieved when adding topic context and enriching the DEG_Twitter_ with features from DEG_SIder_

A training set of 1,390 randomly chosen edges in *DEG*_*Twitter*_ (half of the entire set) was used for cross validation and parameter tuning. The remaining edges composed the test set. Figure 7 summarizes the results. The baseline model demonstrated good performance on our dataset with an *F1* value of .74 in the test set. Including the sentiment context in the model provided minor improvement in both precision and recall values in both the training and test sets. This led to an increased F1 score in both sets. The inclusion of the topic model features provides a significant gain in precision, with an improvement [6 %] over the baseline in both the training and test sets. However, it provides negligible effects on the recall values. Enriching the features set further with the topological features from *DEG*_*Sider*_ shows gains in recall with an increase [7 %] over the sentiment and topic feature sets. These evaluations show that the best performing model is the *DEG*_*Sider*_ enriched model with sentiment and topic context features with an *F1* score of .81 in the test set with a gain [7 %] over the baseline model. This result illustrates several points. Not only does the content of the discussions around medical entities on Twitter help differentiate the relationship, this content can be approximated through sentiment analysis and topic modelling. Additionally, these results point to the value of the DEG model’s ability to transfer information between DEGs through edge matching.

### Discovering unrepresented ADEs

*DEG*_*Sider*_ was used to hypothesize ADEs that were not present in the SIDER database. Here we investigate whether the inherent topology of the DEG can imply the presence of unrepresented information. SIDER is mined from FDA package inserts, however, for various reasons, the data in SIDER may not reflect the current state of ADE knowledge. By enumerating and classifying edges in DEG_Sider_ then mining Twitter feeds and medical literature for supporting evidence, we found several unrepresented ADEs. Table 2 shows the ADE’s that were hypothesized by *DEG*_*Sider*_ along with the text from Twitter posts indicating both the drug and effect. Both tweets were posted by the same user within the 20 day window. Additionally, a reference from the medical literature is provided that confirms awareness of the ADE in the medical community.

**Table 2:** ADEs Discovered through DEG Analysis

Drug	Effect	Ref	Drug Tweet	Effect Tweet
Pravastatin	Eczema	[38]	Sertraline and Propranolol in the morning low dose Propranolol throughout the day Trazodone, Loxapine and **pravastatin** at night	then theres the Clobetasol Propionate for my **Eczema**.
Atenolol	Cramps	[39]	I’m only way to changing my BP medication. The **Atenolol** I have been taking hasn’t been effective enough. I was in hospital last weekend.	I’m just here trying to deal with **cramps** while watching Jesus documentaries on TV.
Vyvanse	Alopecia (Balding)	[40]	Starting to take **Vyvanse** again tomorrow	Heck yeah I went **bald**
Digoxin	Exhaustion	[41]	**digoxin** why u gotta be so complicated?	Why am I so **exhausted** 24/7 I’m so over this ughhhhh
Tamoxifen	Stroke	[42]	I learned about **tamoxifen** and **strokes** after my aunt died of a massive bleed after unnecessary long tamoxifen treatment. Nurse told me!	[see Drug tweet]

Table 2 This table contains ADEs that were implied by the topology of DEG_Sider_. Evidence for each of these ADEs was found both in the Twitter-stream as well as in medical literature. Twitter text is provided with the medical entities in bold (text is nominally altered/censored where appropriate). This process of ADE hypothesis has potential use in helping fill gaps in the databases like SIDER, when evidence from the literature is present. It may also be used to hypothesize new and unreported ADEs for further investigation

This process of hypothesis and evidence gathering has two potential uses. First, it can be used to help identify gaps in SIDER as well as other side effect databases and assist in filling them in with reports from the medical literature. Second, it can be used to hypothesize unknown ADEs for further investigation.

### Synopsis and findings

The experiments in this section support the effectiveness of representing drug and drug-induced effects using the proposed network-based DEG representation. This conclusion follows from the success in differentiating the adverse and pseudo-non-adverse edges found in the two datasets under investigation. The classification performance was better on the *DEG*_*Sider*_, likely because it is built from a relatively clean and curated data source. The noisy environment of Twitter that was used to build *DEG*_*Twitter*_ did indeed impact performance adversely. However, we found that by enhancing the model with features native to Twitter and unavailable to SIDER, namely the semantic context, we were able to improve the model’s performance. Additionally, the consistent DEG structure employed by both *DEG*_*Sider*_ and *DEG*_*Twitter*_ allowed for an information enrichment of *DEG*_*Twitter*_ through edge matching. Thus, we observed the best performance of the *DEG*_*Twitter*_ model when it included the semantic context features as well as information enrichment.

We also found that through our DEG approach, it is possible to hypothesize ADEs that are implied by the DEG structure by enumerating the possible edges and classifying them individually. That we were able to find instances of several of these hypothesized ADEs in the Twitter-stream and medical literature underscores both the efficacy of our DEG model and the value of Twitter as a resource for ADE identification.

## Discussion

The results from our experiments show that by observing temporally related drug and effect terms we are able to extract signal from the twitter stream. This signal, by its nature, represents effects that are correlative, rather than positing causal relationship. It is true that Twitter users makes explicit statements such as “Advil gave me a horrible rash” where they ascribe a putative causal relationship between the drug and effect. However, without rigorous biological investigations of the mechanisms of drug action, including clinical trials, an actual causal relationship cannot be established.

Social media is also a notoriously noisy data source and the provenance of this noise should be recognized and carefully considered both in the design of methods and analysis of data. The most immediate source of noise in our work is the fact that not all drug and effect terms are expressed in the first person perspective, that is, as the personal experience of the user. For example, the text “*My husband was prescribed Lipitor*” does describe an experience, but not the experience of the user. This only becomes a problem when there is a mismatch between the contexts of the drug and effect terms. It should be noted that even when a drug and an effect are mentioned in the same tweet, they may have different contexts. For example, the text “*My husband is loud when he drinks alcohol and he is giving me a headache*.” To estimate the significance of this effect, we sampled 3,077 tweets from the 37,000 tweets used to construct the *DEG*_*Twitter*_. These tweets were manually labeled as either first person experiences or not first person experiences. We found that a strong majority of the tweets did express first person experiences at 77 %. Note that this number constitutes a lower bound on the number of relevant tweets because a tweet involving a third-person reference such as “*My husband took Celexa and is now experiencing exhaustion*” contains a valid drug – effect relationship yet it is not a first person experience. In future work, it will be beneficial to design an additional tweet filter that can deconvolute the two cases potentially focusing on proper noun and pronoun use.

**Fig. 8:**
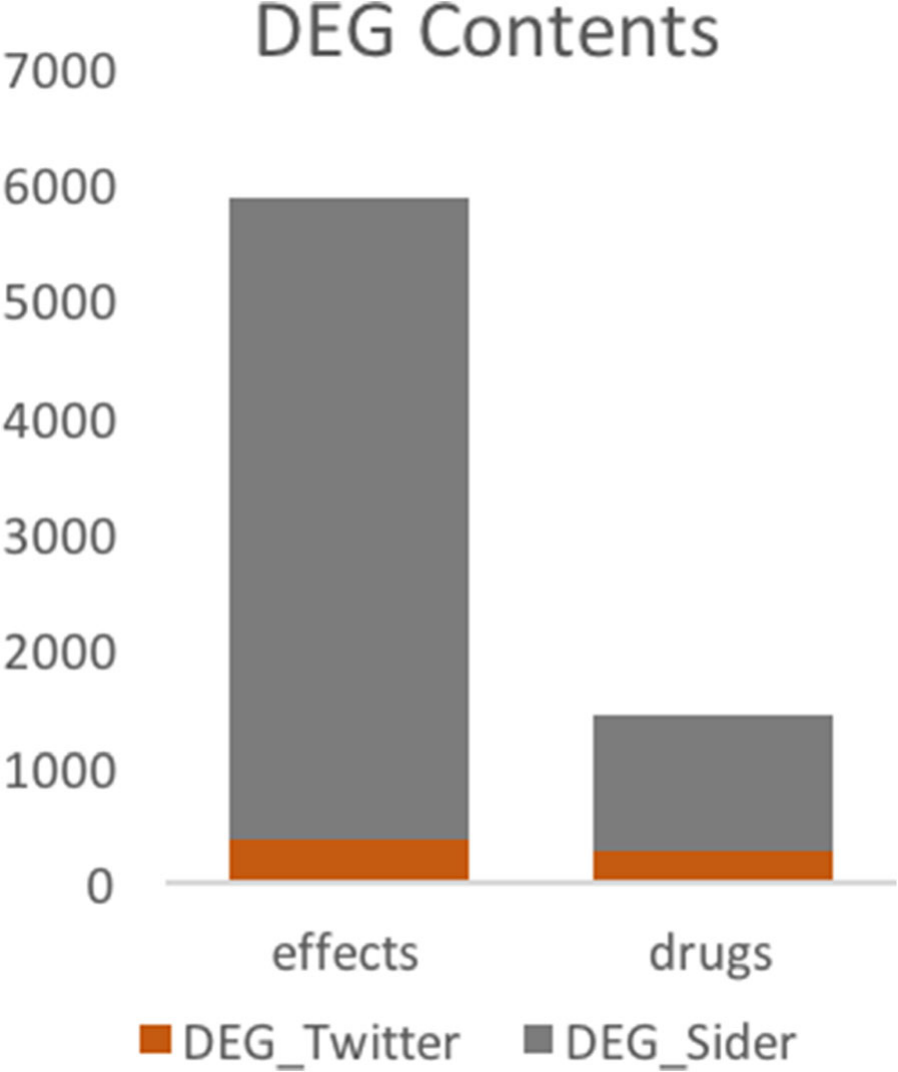
Number of unique drugs and unique effects in DEG_Twitter_ and DEG_Sider_

An additional source of bias in using social media for ADE analysis is that the set of drugs and side effects is limited to what users can articulate. User language is often less precise, less rigorous in usage, and less descriptive than language used in more formal settings resulting in a smaller overall set of drugs and side effects and an intrinsic bias. The limited coverage of drugs and effects found on Twitter is precisely the reason that we sought to augment it with the more complete and refined data found in the SIDER database. Even with the incorporation of SIDER data into the model, the fact remains that many ADEs may simply not be represented in Twitter streams. Figure 8 shows the relative size of *DEG*_*Sider*_ and *DEG*_*twitter*_ indicating the lack of coverage of drug and effect data on Twitter. It would certainly be a worthwhile endeavor in future work to perform a thorough evaluation of the drugs and effects found on Twitter and other social media platforms.

## Conclusions

In this paper, we have described a framework based on graph-theoretic modeling of drug-effect relationships drawn from various data sources. We applied this framework to two sources: SIDER, which is explicit in its purpose of containing these relationships, and Twitter, whose wide range of content has been shown to contain these relationships. The DEG model is general enough to accommodate these diverse data sources, yet rigorous enough to provide a strong mechanism for classification. We built classification models based on *DEG*_*Sider*_ and *DEG*_*Twitter*_ both showed strong performance. The DEG model also allows for the incorporation of domain specific information into the model, such as the semantic context provided by Twitter, which gave significant performance boosts when added to the model. Moreover, the information enrichment ability inherent in the DEG structure allowed *DEG*_*Twiiter*_ to benefit from the strengths of *DEG*_*Sider*_.

Our results further underscore the value real-time information exchange platforms like Twitter’s bring to the field of pharmacovigilance. With a relatively straightforward preprocessing pipeline of tweet filtering and information extraction using freely available tools, a set of drugs and side effects can be extracted from the otherwise noisy stream of Twitter posts. We used the proposed DEG model to predict ADEs that were missing from the SIDER database, and in several cases, we were able to find evidence of these predicted ADEs in the Twitter-stream with further corroboration in the medical literature. This result highlights the potential and value of our method as a tool in the hunt for unknown ADEs.

## Additional file

Additional file 1: Table S1. The list of common drug names used as an initial filter for the Twitter-stream. **Table S2**. Terms that frequently caused erroneous mappings from the MetaMap entity extraction system. (DOCX 23 kb)

## Abbreviations

ADE: Adverse drug event/effect
API: Application program interface
DEG: Drug effect graph
EMR: Electronic medical record
SRS: Spontaneous reporting system
