# Supplementary material for: Leveraging graph topology and semantic context for pharmacovigilance through twitter-streams
Source: BMC Bioinformatics. 2016 Oct 6;17(Suppl 13):335. doi: 10.1186/s12859-016-1220-5 (PMC5073861; doi:10.1186/s12859-016-1220-5)
Supplement: Additional file 1: Table S1. — The list of common drug names used as an initial filter for the Twitter-stream. Table S2. Terms that frequently caused erroneous mappings from the MetaMap entity extraction system. (DOCX 23 kb) [file 12859_2016_1220_MOESM1_ESM.docx]

Supplemental S1.

Table S1: Drug List used to seed the Twitter Streaming API.

| Chemical Name | Common Trade Name | Chemical Name | Common Trade Name |
| --- | --- | --- | --- |
| Levothyroxine | Synthroid | Diltiazem | Cardizem |
| Hydrocodone | [Generic Only] | Insulin Glargine | Lantus |
| Amoxicillin | Amoxil | Thyroid | Armour Thyroid |
| Lisinopril | Prinivil | Bupropion | Wellbutrin |
| Esomeprazole | Nexium | Cetirizine | Zyrtec |
| Atorvastatin | Lipitor | Topiramate | Topamax |
| Simvastatin | Zocor | Valacyclovir | Valtrex |
| Clopidogrel | Plavix | Eszopiclone | Lunesta |
| Montelukast | Singulair | Acyclovir | Zovirax |
| Rosuvastatin | Crestor | Cefdinir | Omnicef |
| Metoprolol | Lopressor | Clindamycin | Cleocin |
| Escitalopram | Lexapro | Colchicine | Colcrys |
| Azithromycin | Zithromax | Gemfibrozil | Lopid |
| Albuterol | ProAir HFA | Guiafenesin | Robitussin |
| Hydrochlorothiazide | HCTZ | Glipizide | Glucotrol |
| Metformin | Glucophage | Irbesartan | Avapro |
| Sertraline | Zoloft | Metoclopramide | Reglan |
| Ibuprofen | Advil | Losartan | Cozaar |
| Zolpidem | Ambien | Meclizine | Dramamine |
| Furosemide | Lasix | Metronidazole | Flagyl |
| Omeprazole | Prilosec | Vitamin D | Caltrate |
| Trazodone | Desyrel | Testosterone | AndroGel |
| Valsartan | Diovan | Ropinirole | Requip |
| Tramadol | Ultram | Risperidone | Risperdal |
| Duloxetine | Cymbalta | Olopatadine | Patanol |
| Warfarin | Coumadin | Moxifloxacin | Avelox |
| Amlodipine | Norvasc | Dexmethylphenidate | Focalin |
| Oxycodone | Percocet | Enoxaparin | Lovenox |
| Quetiapine | Seroquel | Fentanyl | Duragesic |
| Promethazine | Phenergan | Dicyclomine | Bentyl |
| Fluticasone | Flonase | Bisoprolol | Zebeta |
| Alprazolam | Xanax | Atomoxetine | Strattera |
| Clonazepam | Klonopin | Ramipril | Altace |
| Benazepril | Lotensin | Temazepam | Restoril |
| Meloxicam | Mobic | Phentermine | Adipex P |
| Citalopram | Celexa | Quinapril | Accupril |
| Cephalexin | Keflex | Sildenafil | Viagra |
| Tiotropium | Spiriva | Ondansetron | Zofran |
| Gabapentin | Neurontin | Oseltamivir | Tamiflu |
| Aripiprazole | Abilify | Methotrexate | Rheumatrex |
| Potassium | K-Tab | Dabigatran | Pradaxa |
| Cyclobenzaprine | Flexeril | Budesonide | Uceris |
| Methylprednisolone | Medrol | Doxazosin | Cardura |
| Methylphenidate | Concerta | Desvenlafaxine | Pristiq |
| Fexofenadine | Allegra | Insulin Lispro | Humalog |
| Carvedilol | Coreg | Clarithromycin | Biaxin |
| Carisoprodol | Soma | Buspirone | Buspar |
| Digoxin | Lanoxin | Finasteride | Proscar |
| Memantine | Namenda | Ketoconazole | Nizoral |
| Atenolol | Tenormin | Solifenacin | VESIcare |
| Diazepam | Valium | Methadone | Dolophine |
| Oxycodone | OxyContin | Minocycline | Minocin |
| Risedronate | Actonel | Phenazopyridine | Pyridium |
| Folic Acid | Folvite | Spironolactone | Aldactone |
| Olmesartan | Benicar | Vardenafil | Levitra |
| Prednisone | Deltasone | Clobetasol | Clovate |
| Doxycycline | Vibramycin | Benzonatate | Tessalon |
| Alendronate | Fosamax | Divalproex | Depakote |
| Pantoprazole | Protonix | Dutasteride | Avodart |
| Tamsulosin | Flomax | Febuxostat | Uloric |
| Triamterene | Dyazide | Lamotrigine | Lamictal |
| Paroxetine | Paxil | Nortriptyline | Pamelor |
| Buprenorphine | Suboxone | Roflumilast | Daliresp |
| Enalapril | Vasotec | Rabeprazole | Aciphex |
| Lovastatin | Mevacor | Etanercept | Enbrel |
| Pioglitazone | Actos | Nebivolol | Bystolic |
| Pravastatin | Pravachol | Nabumetone | Relafen |
| Fluoxetine | Prozac | Nifedipine | Procardia |
| Insulin Detemir | Levemir | Nitrofurantoin | Macrobid |
| Fluconazole | Diflucan | Nitroglycerine | NitroStat SL |
| Levofloxacin | Levaquin | Oxybutynin | Ditropan |
| Rivaroxaban | Xarelto | Tadalifil | Cialis |
| Celecoxib | Celebrex | Triamcinolone | Kenalog |
| Codeine | Tylenol #2 | Rivastigmine | Exelon |
| Mometasone | Nasonex | Lansoprazole | Prevacid |
| Ciprofloxacin | Cipro | Cefuroxime | Ceftin |
| Pregabalin | Lyrica | Methocarbamol | Robaxin |
| Insulin Aspart | Novolog | Travoprost | Travatan |
| Venlafaxine | Effexor | Lurasidone | Latuda |
| Lorazepam | Ativan | Terazosin | Hytrin |
| Ezetimibe | Zetia | Sumatriptan | Imitrex |
| Estrogen | Premarin | Raloxifene | Evista |
| Allopurinol | Zyloprim | Mirtazepine | Remeron |
| Penicillin | Pen VK | Adalimumab | Humira |
| Sitagliptin | Januvia | Benztropine | Cogentin |
| Amitriptyline | Elavil | Baclofen | Gablofen |
| Clonidine | Catapres | Hydralazine | Apresoline |
| Latanoprost | Xalatan | Mupirocin | Bactroban |
| Lisdexamfetamine | Vyvanse | Propranolol | Inderal |
| Niacin | Niaspan | Varenicline | Chantix |
| Naproxen | Aleve | Verapamil | Verelan |
| Dexlansoprazole | Dexilant | Clotrimazole | Lotrimin |
| Glyburide | Diabeta | Phenytoin | Dilantin |
| Olanzapine | Zyprexa | Liraglutide | Victoza |
| Tolterodine | Detrol | Ticagrelor | Brilinta |
| Ranitidine | Zantac | Diclofenac | Voltaren |
| Famotidine | Pepcid | Saxagliptin | Onglyza |
| Tizanidine | Zanaflex | Lomitapide | Juxtapid |

Table S2: Erroneous, irrelevant MetaMap mappings

| **MetaMap Mapping** | **Twitter Text** |
| --- | --- |
| Altretamine | hmm |
| Megestrol | meg |
| acetohydroxamic acid | aha |
| Punctate Inner Choroidopathy | pic |
| Benzaldehyde Dimethane Sulfonate | ben |
| Zanamivir | gana |
| Toremifene | tor |
| Fluorouracil | fu |
| Fluorides | f |
| FML | fml |
| temozolomide | tmz |
| SHH protein human | shh |
| Polyvinyl Chloride | vinyl |
| Proline | pro |
| Methamphetamine | chalk |
| Methamphetamine | tina |
| Dimethyl Sulfoxide | dmso |
| Vidarabine | ara a |
| Remid | remid |
| Compro | compro |
| P-2 | p 2 |
| S-2 | s 2 |
| Versed | vers |
| Tretinoin | tra |
| Pentoxifylline | ptx |
| Halotestin | hal |
| Halotestin | hals |
| Monarch | monarch |
| Ifosfamide | iff |
| Ifosfamide | ifo |
| SSD | ssd |
| adrenomedullin | adm |
| Orosomucoid | agp |
| Deoxyuridine | du |
| Vincristine | lcr |
| Azathioprine | aza |
| Busulfan | bsf |
| Busulfan | bu |
| Vals | vals |
| aminosalicylic acid | pas |
| Ethacrynic Acid | ea |
| THBD protein human | tm |
| Progesterone | prg |
| Alli | alli |
| Rifampin | rif |
| Encore | encore |
| Spironolactone | spl |
| Furosemide | frs |
| SHH protein human | shh |
| Polyvinyl Chloride | vinyl |
| Valine | val |
| Acetaminophen | apap |
| Aspirin | asa |
| Gentamicin Sulfate (USP) | gm |
| Prompt | prompt |
| Hydrocortisone | hc |
| refresh | refresh |
| refresh | refreshing |
| Levothyroxine Sodium | levothyroxine sodium |
| Silver preparation | silver |
